# Measurement properties of the Sexual Risk Behaviors Scale (SRBS) in university students in Brazil

**DOI:** 10.31744/einstein_journal/2026AO1714

**Published:** 2026-03-30

**Authors:** Jainé Dalmolin, Guilherme Tavares de Arruda, Erisvan Vieira da Silva, Caroline Arboite de Fraga, Amanda dos Santos Candido, Hedioneia Maria Foletto Pivetta, Melissa Medeiros Braz

**Affiliations:** 1 Postgraduate Program in Movement Sciences and Rehabilitation Universidade Federal de Santa Maria Santa Maria RS Brazil Postgraduate Program in Movement Sciences and Rehabilitation, Universidade Federal de Santa Maria, Santa Maria, RS, Brazil.; 2 Department of Physiotherapy Universidade Federal de Juiz de Fora Campus Governador Valadares Governador Valadares MG Brazil Department of Physiotherapy, Universidade Federal de Juiz de Fora, Campus Governador Valadares, Governador Valadares, MG, Brazil.; 3 Postgraduate Program in Gerontology Universidade Federal de Santa Maria Santa Maria RS Brazil Postgraduate Program in Gerontology, Universidade Federal de Santa Maria, Santa Maria, RS, Brazil.; 4 Department of Physiotherapy and Rehabilitation Universidade Federal de Santa Maria Santa Maria RS Brazil Department of Physiotherapy and Rehabilitation, Universidade Federal de Santa Maria, Santa Maria, RS, Brazil.

**Keywords:** University students, Sexual health, Sexual behavior, Psychometrics, Risk-taking

## Abstract

**Objective:**

This study aimed to translate and evaluate the measurement properties of the Sexual Risk Behaviors Scale into Brazilian Portuguese for use in university students.

**Methods:**

Content validity was assessed by experts and Brazilian adults. Structural validity was assessed using a confirmatory factor analysis. Internal consistency and test-retest reliability were assessed using McDonald’s Omega and intraclass correlation coefficient (ICC), respectively. Measurement invariance between females and males was evaluated using multigroup confirmatory factor analysis. The measurement error and floor and ceiling effects were also evaluated.

**Results:**

The experts and Brazilian adults suggested that the five-item Sexual Risk Behaviors Scale was more relevant, comprehensive, and comprehensible. In total (n=633; 22.8±2.0 years), 362 (57.2%) women and 271 (42.8%) men participated in the study. Structural validity indicated a one-factor structure as the best-fitting model, and the internal consistency was sufficient (McDonald’s Omega=0.723). There was measurement invariance between females and males. The test-retest reliability was found to be sufficient (ICC=0.873). Moreover, no systematic error were observed. There was a floor effect across the entire sample.

**Conclusion:**

The Sexual Risk Behaviors Scale is valid and reliable for assessing sexual risk behaviors in university students in Brazil.

## HIGHLIGHTS

The Brazilian Portuguese version of the Sexual Risk Behaviors Scale has strong measurement properties, including content validity, structural validity, and internal consistency.

The Sexual Risk Behaviors Scale showed measurement invariance between females and males and sufficient test-retest reliability, supporting its stability and applicability across sexes.

The Sexual Risk Behaviors Scale is a valid and reliable instrument for assessing sexual risk behaviors among university students in Brazil.

## In Brief

Understanding sexual risk behaviors among university students is important to prevent sexually transmitted infections. This study translated the Sexual Risk Behaviors Scale into Brazilian Portuguese and confirmed its validity and reliability for assessing sexual risk behaviors among university students. By providing a quick and effective instrument, the results can support better public health strategies and interventions aimed at promoting safer sexual practices among the Brazilian university population.

## Graphical abstract

**Figure:**
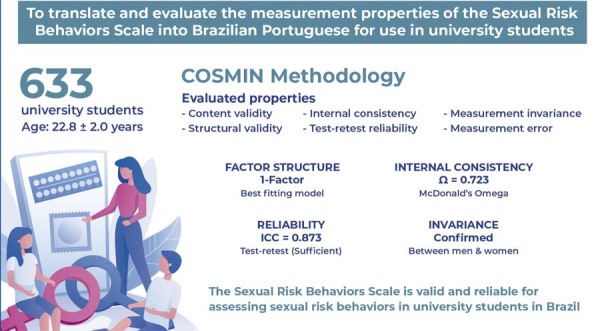

## INTRODUCTION

Sexual risk behaviors (SRBs) are practices that may expose individuals to serious risks to their health and well-being, such as the spread of sexually transmitted infections,^( 1 )^ unintended pregnancy,^( 2 )^ and conflicts in family and social relationships.^( 3 )^ Examples of these behaviors include casual sexual partnerships,^( 4 )^ legal or illegal drug use, early sexual debut, alcohol use, and unprotected sex.^( 3 )^ These behaviors are considered risky because they are associated with negative health outcomes.^( 5 )^ For example, engaging in casual relationships increases the exposure to new sexual partners, often without consistent condom use, which significantly increases the risk of sexually transmitted infections.^( 6 )^ In addition, unprotected sex, whether vaginal or anal, without proper condom use, is one of the main routes of transmission of sexually transmitted infections, especially unprotected anal sex practices that pose an even greater risk.^( 7 )^ The use of legal or illegal drugs, as well as alcohol consumption, increases the likelihood of unplanned sexual intercourse, failure to use contraception, and failure to use protection during sex.^( 8 )^

Among young people, the early onset of sexual life, adoption of various high-risk behaviors, and feelings of invulnerability contribute to engaging in unprotected sex, which is itself a high-risk behavior. This combination has led to a significant increase in sexually transmitted infections, especially HIV, among people aged 20 to 29 years.^( 9 )^

A study on HIV infections in Brazil showed that among young men (15-24 years), the incidence of HIV reached levels of more than 70 per 100,000 population between 2014 and 2016. Among women of the same age group, the incidence rate in 2018 was 18.6 per 100,000 inhabitants.^( 10 )^ Another study conducted in Brazil on men who have sex with men showed an increase in the prevalence of HIV infection in 2016 (18.4%).^( 11 )^ The authors suggested that this increase may be related to deficiencies in prevention efforts, such as a decrease in condom promotion and distribution.

The identification of SRBs is fundamental for health professionals in disease protection and prevention^( 12 )^ as it improves the assessment of more exposed populations and health care.^( 3 )^ In this sense, health assessment tools, such as patient-reported outcome measures (PROMs), become necessary because they allow the identification of at-risk individuals through their own responses.^( 13 )^ For this reason, the Sexual Risk Behavior Scale (SRBS) was developed to measure SRBs in university students.^( 3 )^ This PROM has six items scored on a 5-point Likert scale, with a one-factor structure, sufficient internal consistency (Cronbach’s alpha=0.76), and invariance between women and men and across sexual orientation (heterosexual vs. non-heterosexual) in the United Kingdom.^( 3 )^ Unlike previous sexual risk assessments that have generally focused on unprotected sex,^( 13 )^ the SRBS emphasizes the frequency of specific risky sexual practices, such as unprotected anal sex, rather than simply counting the total number of sexual partners.^( 3 )^

Although previous scales^( 12 , 14 )^ have provided important insights into sexual risk by assessing a variety of behaviors and predisposing factors, they have not adequately captured all the critical aspects. The SRBS fills an important gap by emphasizing specific behaviors, such as sex with casual partners and the use of alcohol and other substances before or during sex, and shifting the focus away from condom use.^( 3 )^ Furthermore, compared with other existing sexual risk scales,^( 13 )^ one of the main advantages of the SRBS is its shortness and sufficient measurement properties.

Given the worldwide incidence of sexually transmitted infections^( 15 )^ and the increase in alcohol consumption in Brazil,^( 16 )^ SRBs represent an important public health problem.^( 17 )^ The use of measurement instruments to assess SRBs is essential to identify and appropriately address sexual health problems,^( 13 )^ especially in Brazil, where there are no specific tools for this assessment. The SRBS appears to be an easy-to-understand and quick scale for assessing SRBs in university students.^( 3 )^

## OBJECTIVE

This study aimed to translate the Sexual Risk Behaviors Scale into Brazilian Portuguese and evaluate its measurement properties in university students.

## METHODS

### Design

This cross-sectional, online, repeated measures study was reported according to the COnsensus-based Standards for the selection of health Measurement INstruments (COSMIN) recommendations^( 18 )^ and the taxonomy for the following measurement properties: content validity (the degree to which the content of a PROM is an adequate reflection of the construct to be measured), structural validity (the degree to which the scores of a PROM are an adequate reflection of the dimensionality of the construct to be measured), internal consistency (the degree of the interrelatedness among the items), measurement invariance (the degree to which the performance of the items on a translated or culturally adapted PROM are an adequate reflection of the performance of the items of the original version of the PROM), test-retest reliability (the extent to which scores for patients who have not changed are the same for repeated measurement over time), and measurement error (the systematic and random error of a patient’s score that is not attributed to true changes in the construct to be measured).^( 19 )^ Floor and ceiling effects were also assessed.

The study protocol was reviewed and approved by the Research Ethics Committee of *Universidade Federal de Santa Maria* (CAAE: 56633222.5.0000.5346; #5.489.920). All procedures complied with the Declaration of Helsinki and relevant national regulations. Informed consent was obtained electronically from all participants before their participation in the study. All anonymized data generated and analyzed during this study were stored on a secure institutional server and will be preserved for at least 5 years. Access to the datasets is restricted to the research team; however, they may be made available upon reasonable request in compliance with Brazil’s General Data Protection Law. No personally identifiable information will be shared or published.

### Procedures and population

The participants were invited to participate in the study through Facebook^®^, Instagram^®^, WhatsApp^®^, and emails from universities via a Google Forms link. No paid advertisements were used in this study. Brazilian university students aged 18-25 years who could speak, read, and write in Brazilian Portuguese were included. We included individuals in this age group because the SRBS was developed for this population.^( 3 )^ Moreover, we included people of all sexes from different geographic regions of Brazil to increase sociocultural variability. Those who were not university students were excluded.

The sample size for structural validity and measurement invariance was calculated according to Kline’s recommendations:^( 20 )^ 5 to 10 people per item of the PROM; however, more than 100 people were sufficient. Therefore, the minimum sample size required for structural validity was 100. The sample size required for test-retest reliability was calculated using the formula for two repeated measures, α=0.05, power=80%, expected intraclass correlation coefficient (ICC)=0.8, lower limit of acceptable ICC=0.6, and expected dropout rate=20%.^( 21 )^ The total expected sample size was 49.

### Translation and content validity assessment

The translation and content validity of the SRBS were assessed in five steps. First, two independent translators translated the original version of the SRBS into Brazilian Portuguese. Both translators were Brazilian and had no prior knowledge of the SRBS. In the second step, the two translated versions of the SRBS were merged into a single version and independently back-translated into English by two other Native American translators. The two back-translators were not familiar with the original instrument. In the third step, discrepancies between the back-translations were resolved by consensus. In the fourth step, the content validity of the SRBS translated into Brazilian Portuguese was assessed by an expert committee composed of seven professionals who were experts in human sexuality and one professional with experience in studying the measurement properties of measurement instruments. Finally, the content validity of the SRBS translated into Brazilian Portuguese was assessed using the target population and was culturally adapted into Brazilian Portuguese.^( 22 )^

The content validity of the SRBS, translated into Brazilian Portuguese, was assessed by an expert committee and the target population through semi-structured interviews using Google Meet^®^. The interviews were conducted by a trained interviewer using an interview guide that included topics related to the SRBS structure, missing concepts, and suggestions for modifying the PROM. The interviews were recorded, transcribed verbatim, and analyzed by two other researchers. An interviewer asked the expert committee and target population about the relevance and comprehensibility of each item, instructions, recall period, response options, and comprehensiveness of the SRBS items. A spreadsheet was used to control response saturation after the interviews were transcribed. The content of the transcribed interviews was analyzed using content analysis with thematic category coding, and the main categories of concepts and words were described.^( 23 )^

### Test-retest reliability and measurement error

We used 12 to 22 days as the time interval between the test and retest to assess test-retest reliability and measurement error. To ensure that participants had not received any sexual therapy or intervention at the retest, they were asked to answer “yes” or “no” to the question “From the time you first participated in this research to the present, have you received any sexual or sexuality therapy?” Participants who answered “no” to this question on the retest were included in the analyses of test-retest reliability and measurement error. The invitation to participate in the retest was sent by email to participants who provided an email address during the test.

### Sample characterization

Participants were characterized using a questionnaire consisting of the following items: age, region of residence in Brazil (Southeast, Northeast, South, North, or Midwest), biological sex (male or female), gender identity (cisgender, transgender, or non-binary), sexual orientation (heterosexual, gay/lesbian, bisexual, or other), clinician-diagnosed anxiety and/or depression, and relationship (no sexual partner or with sexual partner). For sexual orientation, we included pansexual, asexual, and demisexual in the “other” category.

### Sexual Risk Behavior Scale (SRBS)

The SRBS was developed to assess the frequency of risk of engaging in SRBs in the past month. Items on the SRBS were answered on a scale from zero (never) to four (very often), with higher scores indicating a higher risk of engaging in SRBs. The six items included: “how often you have anal sex without a condom,” “how often you have vaginal sex without a condom,” “oral sex without protection,” “sex while under the influence of alcohol,” “sex while under the influence of drugs or substances,” and “sex without a condom with someone you have just met.” In the development study, the measurement properties of this PROM showed a single factor with sufficient internal consistency (Cronbach’s alpha=0.76) and invariance across sexes.^( 3 )^

### Data analysis

Content validity was assessed by the responses of the expert committee and the target population through content analysis, with selective coding of transcripts and grouping of codes into thematic categories. In the content analysis, we noted the frequency of concepts and words contained in the data. Finally, the categories of concepts and words were described, and participants responded to whether the instrument required revision.^( 23 )^

Structural validity was assessed using a confirmatory factor analysis (CFA). In the CFA, we used Weighted Least Squares with Mean and Variance adjusted as the estimator, Root Mean Square Error of Approximation (RMSEA), Standardized Root Mean Squared Residual (SRMR), Comparative Fit Index (CFI), and Tucker-Lewis Index (TLI). RMSEA and SRMR <0.08 and CFI and TLI >0.90 were considered appropriate models.^( 24 )^ Items with modification indices (MI) >30,000 showed error covariance. Additionally, we used the Bayesian Information Criterion (BIC) and the Sample-size Adjusted Bayesian Information Criterion (SABIC) to compare the one- and two-factor models. The model with the lowest BIC value was considered the most appropriate.^( 25 )^ Internal consistency was assessed using McDonald’s Omega. Values of McDonald’s Omega ≥0.70^( 26 )^ were considered sufficient.

Measurement invariance with Weighted Least Squares with Mean and Variance adjusted as the estimator was assessed using multigroup CFA between females and males according to biological sex. We compared the configural, metric, and scalar levels. For configural invariance, the factorial structure must be similar across groups. In metric invariance, the factor loadings of configural invariance are fixed and indicate the magnitude of the factor loadings across groups. Scalar invariance was evident when the item loadings and intercepts were parallel across groups. Invariance was observed when ΔCFI was ≤0.01 and ΔRMSEA was ≤0.015.^( 27 )^

We assessed test-retest reliability using the ICC_agreement_ with a two-way mixed-effects model with interaction for absolute agreement between mean measurements. An ICC_agreement_ ≥0.7 was considered adequate.^( 28 )^ Measurement error was calculated using the Standard Error of Measurement (SEM^agreement^) [SD^difference^/√2], the Smallest Detectable Change (SDC^agreement^) at the individual level [SEM × 1.96 ×√2],(29) and the Limits of Agreement (LoA) [d-±(1.96 × SD^difference^)]. The d- and SD^difference^ are the mean and standard deviation (SD) of the differences between the test and retest scores, respectively.^( 29 )^

The distribution of the SRBS scores was assessed based on floor and ceiling effects. Floor or ceiling effects of less than 15% were considered appropriate.^( 22 )^ Missing values (sample characterization variables) were excluded from the analyses. Responses to the SRBS were required, and there were no missing values. All analyses were conducted using the lavaan package in R (version 4.2.1; R Core Team, Vienna, Austria) and RStudio version 2024.04 (Posit Software, PBC, Boston, MA, USA).

## RESULTS

### Content validity

The content validity, including face validity, of the SRBS was judged to be adequate by an expert committee, with minor modifications. The expert committee suggested the inclusion of “and/or vaginal sex / e/ou sexo vaginal” and “condom / camisinha” in item 1 (How often have you had vaginal sex without a condom?) to provide broader coverage of the types of sex and to improve comprehension of the term “condom.” Thus, items 1 (How often have you had vaginal sex without a condom?) and 2 (How often have you had anal sex without a condom?) were merged. In addition, the wording of items 2, 3, and 4 was modified to improve comprehension. In the new item 2 [How often have you performed oral sex without protection (condom or dental dam)?], the term “dental dam” was replaced with “condom or other method / preservativo ou outro método” because it is often used instead of “dental dam.” In item 3 [How often have you had sex while under the influence of alcohol (i.e., drunk)?], the term “influence of alcohol (drunk)” was replaced by “influence of alcoholic beverage (drunk) / influência de bebida alcoólica (bêbado).” Moreover, in item 4 (How often have you had sex while under the influence of drugs or substances?), “alter sensations, awareness, and emotional state / alteram as sensações, consciência e estado emocional” was added. After the modifications, a new round was conducted with the expert committee, which judged the modified version of the instrument to be appropriate.

The mean age of participants in the cognitive interview step was 22.4 years (SD=1.7), and the majority were from the southern region of Brazil (n=6; 42.9%), had higher education (n=10; 71.4%), and self-identified as cisgender (n=11; 78.6%). Participants found the SRBS instructions, recall time, items, and response options comprehensive, relevant, and comprehensible. The translation of the SRBS into Brazilian Portuguese were carried out with formal authorization from the original author Emanuele Fino, available in the Table 1S, Supplementary Material.

### Sample characteristics

A total of 362 (57.2%) women and 271 (42.8%) men participated in the study (mean age=22.8, SD=2.0 years). Table 1 presents the participants’ sociodemographic characteristics. In the total sample, most participants lived in Southern Brazil (n=268; 42.3%), were cisgender (n=624; 98.6%), heterosexual (n=314; 49.6%), and had sexual partners (n=355; 56.1%).

**Table 1:** Characteristics of the study participants

	Female (n = 362)	Male (n = 271)	Total sample (n = 633)
Age (years), mean±SD	22.1±2	22.3±1.9	22.8±2
Geographic region^a^, n (%)			
South	166 (45.9)	102 (37.6)	268 (42.3)
Southeast	82 (22.7)	61 (22.5)	143 (22.6)
Midwest	17 (4.7)	15 (5.5)	32 (5.1)
North	21 (5.8)	15 (5.5)	36 (5.7)
Northeast	74 (20.4)	78 (28.8)	152 (24.0)
Gender identity, n (%)			
Cisgender	362 (100)	262 (96.7)	624 (98.6)
Transgender	0	02 (0.7)	02 (0.3)
Non-binary	0	07 (2.6)	07 (1.1)
Sexual orientation^a^, n (%)			
Heterosexual	172 (47.5)	142 (52.4)	314 (49.6)
Gay/lesbian	14 (3.9)	54 (19.9)	68 (10.7)
Bisexual	0	53 (19.6)	53 (8.7)
Other	175 (48.3)	21 (7.8)	196 (31)
Relationship, n (%)			
No sexual partner	120 (33.1)	158 (58.3)	278 (43.9)
With sexual partner	242 (66.9)	113 (41.7)	355 (56.1)
Anxiety, n (%)	145 (40.1)	71 (26.2)	216 (34.1)
Depression, n (%)	69 (19.1)	38 (14)	107 (16.9)

Missing value.

SD: standard deviation.

### Structural validity and internal consistency

For both models tested (one- and two-factor), all items had factor loadings (>0.40). Residual correlations were added to the items with the highest MI in the one-factor model: 3 to 4 (MI=102.71), 1 to 2 (MI=95.02), 1 to 3 (MI=34.95), and 1 to 4 (MI=31.63). According to the BIC and SABIC, the one-factor model was recommended. The final one-factor model [χ²(df)=588.84(10)] showed adequate goodness of fit indices for CFI (0.995), TLI (0.96), SRMR (0.01), and RMSEA (0.06, 90% confidence interval (95%CI) [0.00 - 0.14]). McDonald’s Omega was sufficient for the one-factor model (0.723). Tables 2 and 3 present the item factor loadings and goodness-of-fit indices of the SRBS, respectively.

**Table 2:** Factor loadings and internal consistency for items in the Sexual Risk Behaviors Scale (n = 633)

Items	One-factor model	Two-factors model	
		F1	F2
1. Anal sex and/or vaginal sex without a condom	0.496	0.752	
2. Oral sex without protection	0.593	0.956	
3. Sex while under the influence of alcohol	0.593		0.820
4. Sex while under the influence of drugs or substances	0.419		0.768
5. Sex without a condom with someone you have just met	0.431		0.534

F1: Factor 1. F2: Factor 2.

**Table 3:** Goodness-of-fit indices for the Sexual Risk Behaviors Scale

Adjustment indices	One-factor model	Two-factor model
CFI	0.995	0.984
TLI	0.955	0.959
SRMR	0.013	0.057
RMSEA [90% CI]	0.064 (0.000 - 0.141)	0.093 (0.061 - 0.128)
BIC	9312.206	9351.080
SABIC	9267.757	9300.282
McDonald’s Omega	0.723	F1: 0.710 F2: 0.589

BIC: Bayesian Information Criterion; CFI: Comparative Fit Index; CI: Confidence Interval; RMSEA: Root Mean Square Error of Approximation; SABIC: Sample-size Adjusted Bayesian Information Criterion; SRMR: Standardized Root Mean Squared Residual; TLI: Tucker-Lewis Index; F1: Factor 1; F2: Factor 2.

### Measurement invariance

Table 4 presents the MGCFA indices for the configural, metric, and scalar invariances for females and males. According to the results, scalar invariance was observed (ΔCFI=0.028). Thus, there may be differences in the factor loadings of the SRBS between females and males.

**Table 4:** Multigroup confirmatory factor analysis among females and males for the Sexual Risk Behaviors Scale

Measurement invariance	CFI	RMSEA (90%CI)	ΔCFI	ΔRMSEA (90%CI)
Females versus males				
Configural invariance	0.938	0.118 (0.088 - 0.149)	-	-
Metric invariance	0.923	0.111 (0.085 - 0.138)	0.015	0.007 (-0.003 - 0.011)
Scalar invariance	0.895	0.115 (0.092 - 0.138)	0.028	0.004 (-0.007 - 0.000)

CFI: Comparative Fit Index; ΔCFI: Difference in Comparative Fit Index; CI: Confidence Interval; RMSEA: Root Mean Square Error of Approximation; ΔRMSEA: Difference in Root Mean Square Error of Approximation.

### Floor and ceiling effects

In the current study, the total score for the SRBS was 1.22 (SD=0.82) for the total sample, 1.27 (SD=0.77) for females, and 1.16 (SD=0.88) for males. A floor effect was observed in the total sample, with 17.43% of participants scoring the minimum and 0.20% scoring the maximum.

### Test-retest reliability and measurement error

One hundred and sixty-nine (26.70%) participants responded to the retest. Of these, 143 (84.61%) did not receive any sexual therapy or intervention between the test and retest, and responded to the retest between 12 and 22 days. The test-retest reliability value was considered sufficient (ICC^agreement^=0.87, 95%CI [0.767 - 0.924], and the mean difference (d-) between the test and retest was 1.06 points. For the measurement error, the SEM_agreement_, SDC_agreement_, LoA_inf_, and LoA_sup_ were 1.30 (95%CI [1.09 - 1.52]), 3.65 (95%CI [3.02 - 4.20]), -0.51, and 2.62 points, respectively.

## DISCUSSION

In this study, we translated and evaluated the measurement properties of the SRBS in Brazilian university students. The content and structural validity, internal consistency, test-retest reliability, and measurement error of the SRBS were sufficient to assess SRBs in university students. However, this version of the SRBS showed measurement invariance and floor effects. For content validity, the expert committee suggested minor modifications to items 1, 2, 3, and 4 to improve comprehensibility. After merging item 1 (How often have you had vaginal sex without a condom?) with item 2 (How often have you had anal sex without a condom?), the SRBS had five items, and its instructions, items, response options, and recall period were considered relevant, comprehensive, and comprehensible by the expert committee and university students.

According to the CFA, the SRBS had a one-factor structure with adequate goodness-of-fit indices and sufficient internal consistency. Thus, all items together assessed SRBs in university students. In the SRBS development study^( 3 )^ and the five-item Arabic version,^( 30 )^ this PROM also had a one-factor structure with sufficient internal consistency. However, we found scalar invariance in the SRBS when comparing the PROM structure between males and females. This suggests that there are differences in responses between the sexes. Scalar invariance suggests that the factor loadings of the intercepts may not be equivalent for men and women. This indicates that the groups do not have the same baseline for responding to the items.^( 31 )^

Our study found a floor effect in the SRBS. The floor effect occurs when an assessment instrument is not sufficiently sensitive to capture the differences between participants with the lowest scores, thus limiting the ability to detect variations in the measured construct. This may have occurred because the participants in this study had lower levels of the construct being assessed than the PROM that was developed for measurement. Future studies should review the content of the SRBS to ensure that it includes items that are more sensitive to mild manifestations of the construct, expand the range of possible responses, and, if necessary, develop specific versions for subgroups of the population that present with lower scores (e.g., between men and women).^( 22 )^

To the best of our knowledge, this is the first study to evaluate the test-retest reliability and measurement error of the SRBS. We found sufficient test-retest reliability for the SRBS, indicating that respondents’ scores did not change over time. However, if the ICC values were insufficient, the construct assessed by the PROM could change over time, which would compromise the reliability of the PROM for use in clinical practice and scientific research.^( 32 )^ In terms of measurement error, although the SEM_agreement_ of 1.30 and SDC_agreement_ of 3.65 points are clinically relevant, there is considerable variability in scores between tests that must be accounted for over time. The assessment of measurement errors is important for mitigating both systematic and random errors that may arise from real changes in the construct being measured.^( 22 )^ However, when interpreting these results, it is important to recognize that the accuracy of the measurements may be influenced by unidentified factors that may affect the stability of scores over time.

According to the COSMIN methodology,^( 22 )^ self-reported questionnaires aim to capture patients’ perceptions of their situations. These perceptions are considered essential for assessing constructs such as SRBs. These responses represent the participants’ subjective experiences rather than objective clinical outcomes, which is consistent with the purpose of PROMs. However, as COSMIN emphasizes, potential sources of bias, such as memory lapses and social desirability, should be acknowledged when interpreting the data. Thus, although the reported “actual” situation provides valuable insights into participants’ perceptions, future studies that combine PROMs with objective assessments could strengthen the evidence base further.

This study had some limitations. First, because the survey was conducted online, it may have excluded individuals who did not have access to the Internet or who did not feel comfortable participating in online surveys. Second, there was a prevalence of cisgender, heterosexuality, and people with sexual partners, which may affect the generalizability of the results. However, this may be justified because, according to the Brazilian government,^( 33 )^ university students in Brazil have greater access to the Internet, and there has been a recent increase in the number of people with higher education in the country. Despite its limitations, this study is a pioneering step in assessing the measurement properties of the SRBS in Brazilian university students. Additionally, we followed the COSMIN recommendations,^( 18 )^ which ensured methodological robustness.

## CONCLUSION

The Sexual Risk Behaviors Scale translated into Brazilian Portuguese appears to be a promising patient-reported outcome measures for assessing Sexual Risk Behaviors Scale in university students. However, the patient-reported outcome measures were invariant between females and males. We expect that the Sexual Risk Behaviors Scale will have important applications for use in clinical practice and in the prevention of Sexual Risk Behaviors Scale.

## SUPPLEMENTARY MATERIAL

Table 1S Translation of the SRBS into Brazilian Portuguese

**Table:** 

Sexual Risk Behaviors Scale (SRBS)
Escala de comportamentos sexuais de risco
As perguntas a seguir são sobre a natureza e a frequência de seus comportamentos sexuais no ÚLTIMO MÊS. Por favor, leia as questões com atenção e marque apenas uma caixa para cada questão. Lembre-se de que suas respostas são completamente anônimas.
1. Com que frequência você fez sexo anal e/ou sexo vaginal sem camisinha/preservativo?
☐ Nunca
☐ Raramente
☐ Às vezes
☐ Frequentemente
☐ Muito frequentemente
2. Com que frequência você fez sexo oral sem proteção (preservativo ou outro método)?
☐ Nunca
☐ Raramente
☐ Às vezes
☐ Frequentemente
☐ Muito frequentemente
3. Com que frequência você teve sexo enquanto estava sob influência de bebida alcoólica (bêbado)?
☐ Nunca
☐ Raramente
☐ Às vezes
☐ Frequentemente
☐ Muito frequentemente
4. Com que frequência você teve sexo enquanto estava sob a influência de drogas ilícitas ou substâncias que alteram as sensações, consciência e estado emocional?
☐ Nunca
☐ Raramente
☐ Às vezes
☐ Frequentemente
☐ Muito frequentemente
5. Com que frequência você fez sexo sem camisinha com alguém que acabou de conhecer?
☐ Nunca
☐ Raramente
☐ Às vezes
☐ Frequentemente
☐ Muito frequentemente
